# Supplementary material for: Oligo—Not Only for Silencing: Overlooked Potential for Multidirectional Action in Plants
Source: Int J Mol Sci. 2023 Feb 24;24(5):4466. doi: 10.3390/ijms24054466 (PMC10002457; doi:10.3390/ijms24054466)
Supplement: Supplementary file 1 [file ijms-24-04466-s001.zip › S1mFold 2.3 energy DotPlot Lus10031622 at 37 C.pdf]

Output of boxplot_ng (©)
mfold_util 4.7

Created Thu May 26 09:33:38 2022

Fold of L usitatissimum v1 0|Lus10031622 at 37 C.

δG in Plot File = 12.0 kcal/mol

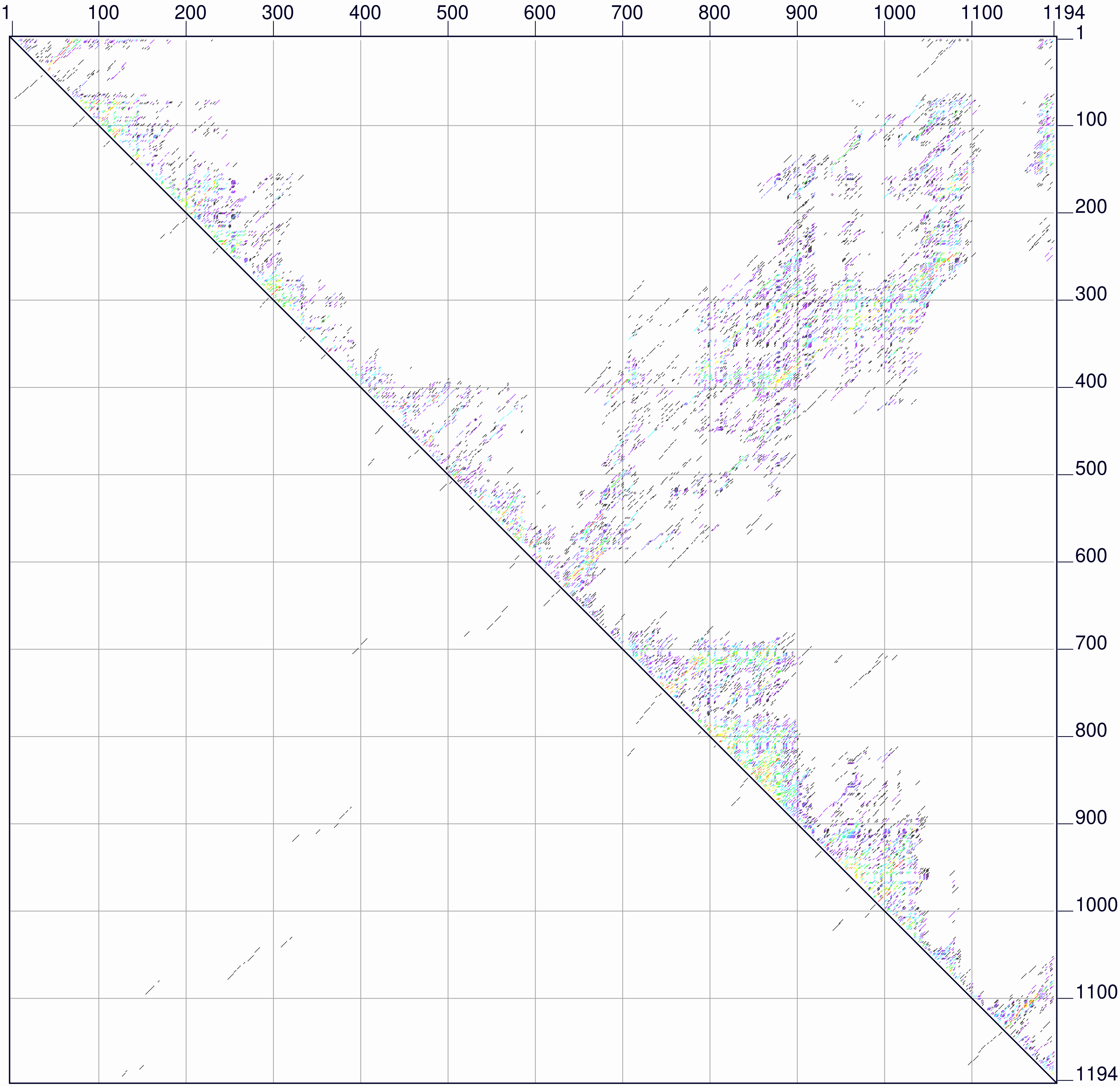

Lower Triangle: Optimal Energy
Upper Triangle Base Pairs Plotted: 28561

Optimal Energy = -371.0 kcal/mol
-371.0 < Energy <= -369.3 kcal/mol
-369.3 < Energy <= -367.6 kcal/mol
-367.6 < Energy <= -365.9 kcal/mol
-365.9 < Energy <= -364.1 kcal/mol
-364.1 < Energy <= -362.4 kcal/mol
-362.4 < Energy <= -360.7 kcal/mol
-360.7 < Energy <= -359.0 kcal/mol
