# Supplementary figures and images for: Oligo—Not Only for Silencing: Overlooked Potential for Multidirectional Action in Plants
Source: Int J Mol Sci. 2023 Feb 24;24(5):4466. doi: 10.3390/ijms24054466 (PMC10002457; doi:10.3390/ijms24054466)

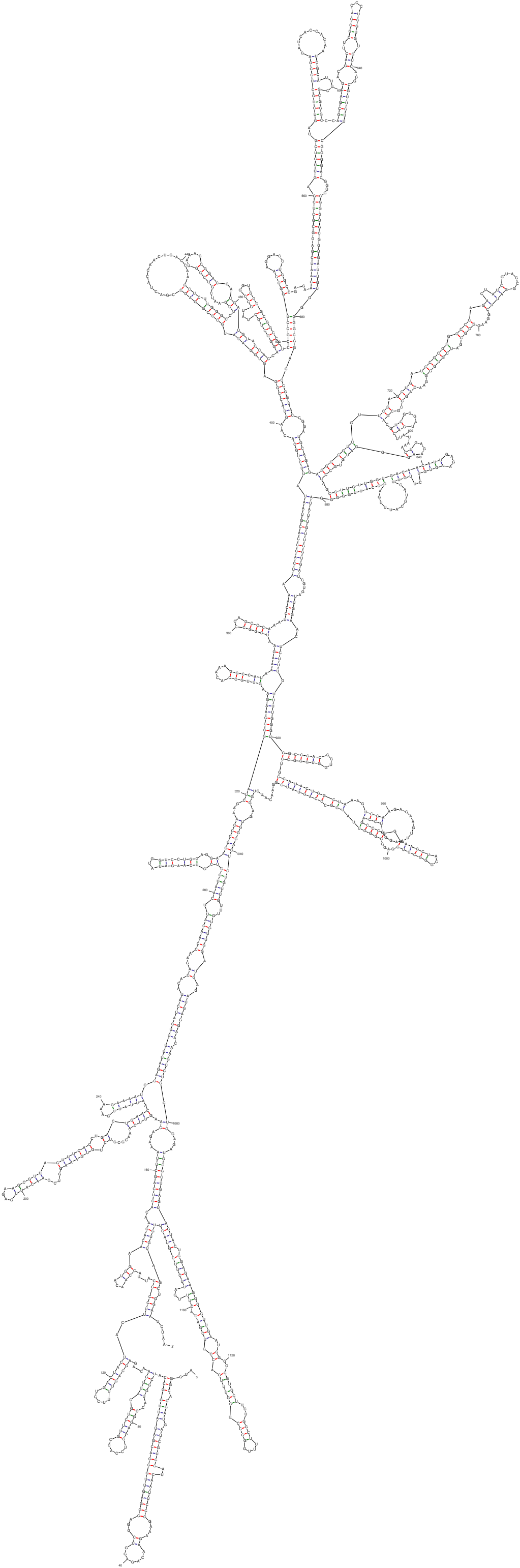

$dG = -371.00$  *L usitatissimum* v1 0|Lus10031622

Supplement: Supplementary file 1 [file ijms-24-04466-s001.zip › S2mFold CHS1 Structure1 37C.pdf]

Probability Profile of Target RNA (from position 1 to 1194)

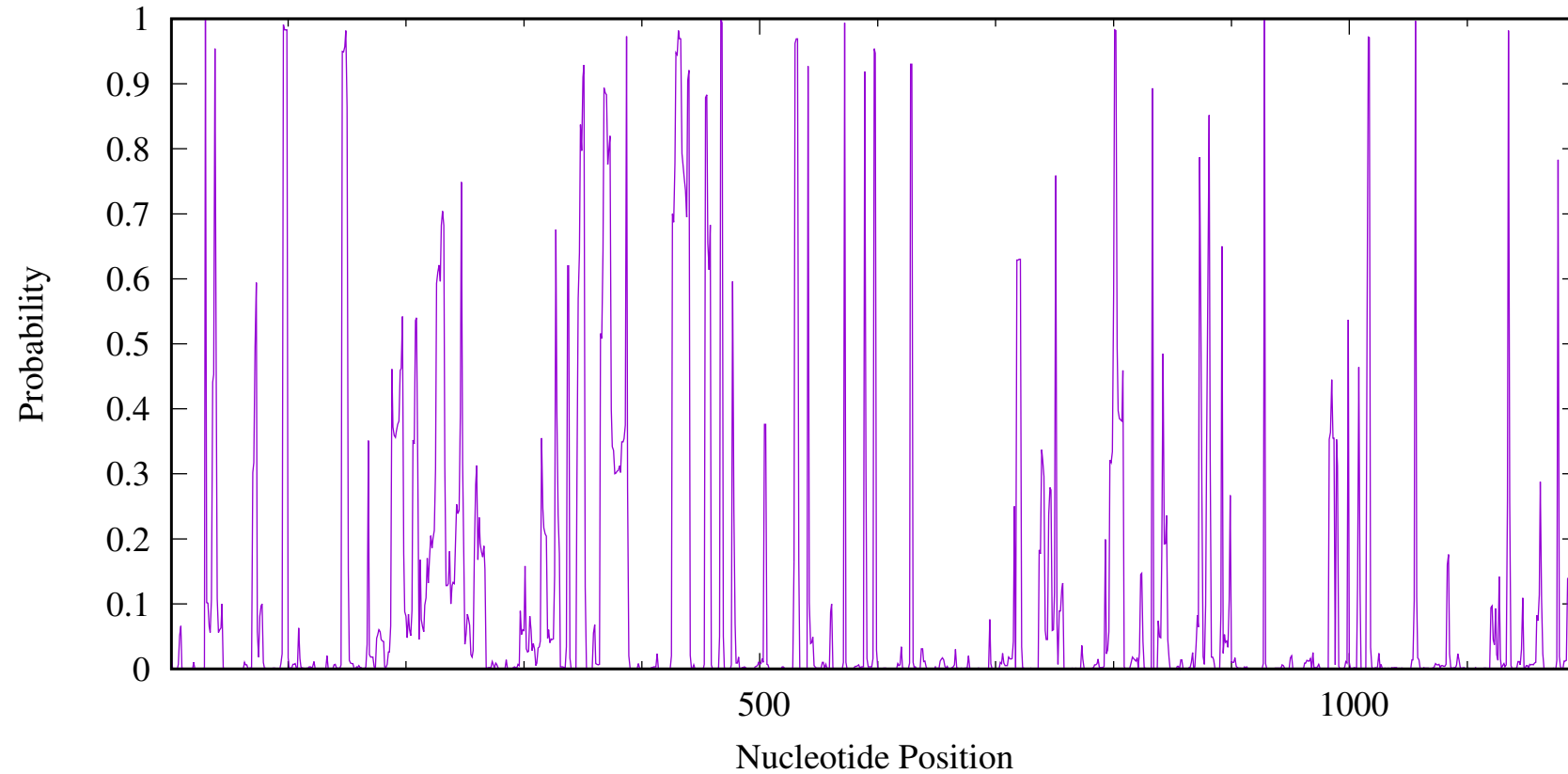

Supplement: Supplementary file 1 [file ijms-24-04466-s001.zip › S3sFold Probability Profile of Target RNA.pdf]
